# Neuralgia, as Pertaining to the Dental Organs

**Published:** 1872-05

**Authors:** N. E. Hollace

**Affiliations:** Boston, Mass.


### AMERICAN JOURNAL

OF

### DENTAL SCIENCE.

Vol. VI. THIRD SERIES--MAY, 1872. No. 1.

### ARTICLE I.

### *Neuralgia, as Pertaining to the Dental Organs.*

BY N. E. HOLLACE, M.D., Boston, Mass.

To this subject we would particularly invite the attention of your readers, as it is one which is most important to all dentists, and which is but little understood.

Certain nerves are the organs of sensation, and like other parts of the body, they are liable to disease. This disease often baffles the efforts of the most diligent and best educated practitioner, to ascertain and remove its cause or to effect a cure. That particular variety known as facial neuralgia arises from pain in the fifth pair of cranial nerves. The fifth pair—tri-facial—(being that from which the greater part of the muscles of the face, and the oral cavity as well as the dental apparatus derive sensation and motion) is most frequently the seat of the difficulty. In most cases ot facial neuralgia the pain of the jaw is at first mistaken for tooth-ache, and frequently ignorant dentists have extracted tooth after tooth, and have at last abandoned the patient to his aggravated suffering.

Hence it will be perceived that the dentist must often be called upon to discriminate between this disease and ordinary tooth-ache, and that unless he be properly informed upon this subject he may add to the terrible suffering of his too confident patient, the additional anguish of tooth extracting and the injury of losing sound and most valuable organs of mastication. The diagnosis of this malady is not difficult, for the pain is generally severe, acute, and more or less lascinating, tingling, burning, itching, &c. There is generally absence of swelling, and great dependence on the sudden changes of the weather; and the fact that it subsides, disappears and returns, will also serve to distinguish it from the continued pain of inflammation.

From tooth ache, depending upon exposed pulp, it may be diagnosed by the evident centralizing of the pain in a certain tooth, by the aggravation of it, when the tooth in fault is struck, and by the positive evidence of a cavity in it. The local causes of facial neuralgia are, first, sensitive dentine; second, irritated pulp; third, exposed pulp; fourth, alveolar abscess; fifth, necrosed bone; sixth, exostosed fangs; seventh, metallic fillings; this (metallic) being a conductor of heat and cold is very apt to cause irritation of the nerve and produce serious results.

I will here mention a case in order to more fully illustrate the importance of thoroughly understanding the local causes of neuralgia, that of Mrs. G. aged about 27 years, of sedentary habits, and naturally rather a nervous temperament. She had for several years been afflicted with the most distressing and painful affection of her left arm, which was pronounced by her physician to be neuralgia caused by indigestion. The pain was sometimes so acute and lancinating that it almost deprived her of reason. It generally commenced near the angle of the shoulder, thence it would dart the whole length of her forearm, and completely deprive her of its use. Her family physican—who by the way is a "regular" and stands high among his medical friends—never made an examination of the mouth to see if he could discover the cause, and if so remove it; but continued to treat his patient in the general way without the slightest relief. Her husband called and informed me that his wife was suffering from neuralgia of her arm and could not get relief. I asked him to bring her to my office and I would examine her teeth. He did so, and upon close examination I found an amalgam filling in the left superior dens sapientia, which I informed her was the cause of her suffering. I tried to persuade the lady to permit me to extract this tooth, which I suspected to be the real cause; she, however, declined until she could consult her family physician. This she did, and his answer was: "What an absurd idea, that a tooth well filled and in a healthy condition should produce neuralgic pains."

Mrs. G. continued to suffer for a few weeks longer; she then came to me and I extracted the tooth. She was relieved within twenty four hours, and is now enjoying perfect health.

This among the many cases which I might mention, will suffice to prove that inasmuch as inflammation and ulceration of the uterus will produce distressing disturbance of the dental organs, so in fact will the dental organs if in a morbid condition produce neuralgic pains in the most remote parts of the body.

---

